# miR-375 induces docetaxel resistance in prostate cancer by targeting *SEC23A* and *YAP1*

**DOI:** 10.1186/s12943-016-0556-9

**Published:** 2016-11-10

**Authors:** Yuan Wang, Rachel Lieberman, Jing Pan, Qi Zhang, Meijun Du, Peng Zhang, Marja Nevalainen, Manish Kohli, Niraj K. Shenoy, Hui Meng, Ming You, Liang Wang

**Affiliations:** 1 Key Laboratory of Hydrobiology in Liaoning Province’s Universities, Dalian Ocean University, Dalian, 116021 China; 2 Department of Pathology, Medical College of Wisconsin, Milwaukee, WI 53226 USA; 3 Department of Pharmacology and Toxicology, Medical College of Wisconsin, Milwaukee, WI 53226 USA; 4 Department of Oncology, Mayo Clinic, Rochester, MN 55905 USA

**Keywords:** Prostate cancer, miR-375, Docetaxel resistance, *SEC23A*, *YAP1*

## Abstract

**Background:**

Treatment options for metastatic castrate-resistant prostate cancer (mCRPC) are limited and typically are centered on docetaxel-based chemotherapy. We previously reported that elevated miR-375 levels were significantly associated with poor overall survival of mCRPC patients. In this study, we evaluated if miR-375 induced chemo-resistance to docetaxel through regulating target genes associated with drug resistance.

**Methods:**

We first compared miR-375 expression level between prostate cancer tissues and normal prostate tissues using data from The Cancer Genome Atlas (TCGA). To examine the role of miR-375 in docetaxel resistance, we transfected miR-375 using a pre-miRNA lentiviral vector and examined the effects of exogenously overexpressed miR-375 on cell growth in two prostate cancer cell lines, DU145 and PC-3. To determine the effect of overexpressed miR-375 on tumor growth and chemo-resistance in vivo, we injected prostate cancer cells overexpressing miR-375 into nude mice subcutaneously and evaluated tumor growth rate during docetaxel treatment. Lastly, we utilized qRT-PCR and Western blot assay to examine two miR-375 target genes, *SEC23A* and *YAP1*, for their expression changes after miR-375 transfection.

**Results:**

By examining 495 tumor tissues and 52 normal tissues from TCGA data, we found that compared to normal prostate, miR-375 was significantly overexpressed in prostate cancer tissues (8.45-fold increase, *p* value = 1.98E-23). Docetaxel treatment induced higher expression of miR-375 with 5.83- and 3.02-fold increases in DU145 and PC-3 cells, respectively. Interestingly, miR-375 appeared to play a dual role in prostate cancer proliferation. While miR-375 overexpression caused cell growth inhibition and cell apoptosis, elevated miR-375 also significantly reduced cell sensitivity to docetaxel treatment in vitro, as evidenced by decreased apoptotic cells. In vivo xenograft mouse study showed that tumors with increased miR-375 expression were more tolerant to docetaxel treatment, demonstrated by greater tumor weight and less apoptotic cells in miR-375 transfected group when compared to empty vector control group. In addition, we examined expression levels of the two miR-375 target genes (*SEC23A* and *YAP1*) and observed significant reduction in the expression at both protein and mRNA levels in miR-375 transfected prostate cancer cell lines. TCGA dataset analysis further confirmed the negative correlations between miR-375 and the two target genes (*r* = −0.62 and −0.56 for *SEC23A* and *YAP1*, respectively; *p* < 0.0001).

**Conclusions:**

miR-375 is involved in development of chemo-resistance to docetaxel through regulating *SEC23A* and *YAP1* expression. Our results suggest that miR-375 or its target genes, *SEC23A* or *YAP1*, might serve as potential predictive biomarkers to docetaxel-based chemotherapy and/or therapeutic targets to overcome chemo-resistance in mCRPC stage.

**Electronic supplementary material:**

The online version of this article (doi:10.1186/s12943-016-0556-9) contains supplementary material, which is available to authorized users.

## Background

Prostate cancer (PC) is the most common non-skin cancer and the second leading cause of cancer- related mortality in men, with >220,000 newly diagnosed cases and 27,000 deaths annually in the United States [1]. Current guidelines recommend that treatments should be considered based on tumor stage. Surgery and radiation therapy are generally effective for early and localized cancers [2]. Docetaxel is used as a standard first-line standard chemotherapy and has shown a survival advantage in metastatic castration-resistant PC (mCRPC), with a median survival advantage of 2 to 3 months when compared to mitoxantrone and improved quality of life [3, 4]. However, a substantial proportion of men with mCRPC do not benefit from docetaxel and those who do benefit invariably progress and die from the disease. Additionally, there has been a considerable expansion in the therapeutic armamentarium with several novel options becoming available for treating mCRPC stage. This has helped prolong life, but has also made clinical decision making complex in choosing between different options. In this study we attempted to characterize molecular mechanisms for development of resistance to docetaxel therapy in PC patients.

miRNAs are a conserved class of small noncoding RNAs that have been recognized as key regulators of gene expression at post-transcriptional level [5, 6]. They are broadly involved in tumor proliferation, invasion, angiogenesis, and drug resistance [5–8]. In recent studies, miRNAs including miR-148a [8], miR-200c [9], miR-205 [2, 9], miR-21 [10], miR-31 [2], and miR-34 [11], have been reported to regulate drug resistance in PC. Characterization of miRNA involvement likely facilitate predictive and prognostic markers of treatments as well as molecular targets for drug development in this stage and tumor type. miR-375 expression in tissue or circulation has been shown to potentially serve as a biomarker for PC diagnosis or prognosis [12–14]. We recently showed significant association of elevated miR-375 levels in plasma with poor overall survival of mCRPC patients [13]. However, this finding is contradictory with other report showing tumor suppressor activity of miR-375 in multiple tumor types by targeting key oncogenes [15]. Although upregulated in primary PC, miR-375 is significantly downregulated in multiple other tumors [15, 16]. To date, the biological role and mechanisms of action of miR-375 in chemotherapy response of mCRPC are not fully understood. A recent study in cervical cancer showed that miR-375 is associated with paclitaxel treatment response through regulation of epithelial–mesenchymal transition (EMT), leading to chemo-resistance [17]. This study suggests that miR-375 may be involved in the development of chemo-resistance in PC to commonly used anti-cancer drug, docetaxel.

In the present study, we performed a series of in vitro and in vivo tests and found consistently higher expression levels of miR-375 in PC tissues and docetaxel-treated PC cells. Elevated miR-375 significantly reduced sensitivity of PC cells to docetaxel treatment, as evidenced by reduced apoptotic cells. Moreover, miR-375 overexpression in PC xenograft tumors following lentivirus transfection contributed to development of resistance to docetaxel treatment. Finally, our data demonstrated that miR-375 may confer chemoresistance through reducing the levels of its target genes *SEC23A* and *YAP1*.

## Methods

### Cell culture and chemicals

Human PC cell lines DU145 and PC-3 were purchased from American Type Culture Collection (Manassas, VA, USA), and cultured in RPMI 1640 media with 10 % fetal bovine serum and penicillin and streptomycin (Gibco, Carlsbad, CA, USA). The cell lines were maintained at 37 °C and 5 % CO_2_ in a humidified incubator. Docetaxel was purchased from Cayman Chemical (MI, USA). The cancer cell lines (DU145 and PC-3) were seeded 3 × 10^5^ per well in six-well plates and incubated overnight, and then treated with docetaxel for 72 h. After 72 h of induction, cells were harvested for subsequent analyses.

### Isolation of total RNA and qRT-PCR

Total RNA were extracted from harvested cells using the Zymo Quick-RNA miniprep extraction kit (Zymo Research, CA, USA) according to the manufacturer’s instructions. Quantitative reverse transcription PCR (qRT-PCR) was conducted using the method as described previously^18^. For the detection of *SEC23A* and *YAP1*, 1 μg of total RNA per sample was converted to cDNA using the SuperScript VILO cDNA Synthesis Kit (Invitrogen, Carlsbad, CA, USA). cDNAs were amplified and detected using SYBR Green PCR Kit (Qiagen, Valencia, CA, USA). The *GADPH* was used as endogenous control for mRNA. For detection of the miRNA, the cDNA products were synthesized using miScript Reverse Transcription Kit (Qiagen, Valencia, CA, USA). The primers specific for miR-375 or endogenous control *RNU6B* were purchased from Qiagen. qRT-PCR was performed using miScript SYBR Green PCR Kit (Qiagen). All reactions were run in triplicate on Bio-Rad C1000 thermal cycler (CFX-96 real-time PCR detection systems, Bio-Rad). The fold change of miRNA or mRNA expression was calculated according to the 2^−ΔΔct^ method. The sequences of all primers are given in Table 1.

**Table 1:** Primers for *YAP1* and *SEC23A* quantification

Gene	Primer	sequences (5′–3′)	Annealing Temperature (°C)
*YAP1*	Forward Primer	5′- TCC TTA ACA GTG GCA CCT ATC AC-3′	58
	Reverse Primer	5′- TCA CCT GTA TCC ATC TCA TCC A-3′	
*SEC23A*	Forward Primer	5′- TGC TAG GAA CTG GGC AGA TG-3′	58
	Reverse Primer	5′- AGC TGC CTC CTG GTC AAA AG-3′	
*GADPH*	Forward Primer	5′- CAC CAG GGC TGC TTT TAA CTC-3′	58
	Reverse Primer	5′- GAA GAT GGT GAT GGG ATT TC-3′	

### Transfection with miR-375 mimics and negative controls

For transient transfection, miRNA mimics and inhibitors were transfected into DU145 and PC-3 cells using Lipofectamine RNAiMAX Reagent (Invitrogen, Carlsbad, CA, USA) following the manufacturer’s protocol. The miR-375 mimic and negative control were obtained from Ambion (Life Technologies, Grand Island, NY, USA). The final concentration of miR-375 mimic, and negative control in the transfection system was 200 nM, respectively. After 24 or 48 h, the cells were collected for subsequent flow cytometry, Western blotting and qRT-PCR.

### Lentivirus transduction

To generate miR-375 stable transfectants, PC cell lines (DU145 and PC-3) were transfected with lentiviral expressing vectors, and stable clones were selected. The lentivirus vector, hsa-mir-375 lentivirus or miR-negative control lentivirus, was obtained from Biosettia (San Diego, CA, USA) with a titer of 10^7^ IU/mL. A total of 1 × 10^5^ DU145 or PC-3 cells were plated in 6-well dishes overnight, and 20 μL of the lentivirus diluted in 2 mL of the Opti-MEM medium was treated in the presence of 5 μg/mL of polybrene (Sigma-Aldrich, St Louis, Missouri, USA). After 24 h, the culture medium was replaced by fresh medium and the transduced cells were positively selected by continuous exposure to 2 μg/mL puromycin (Invivogen, San Diego, CA, USA). At 14 days after the selection, >90 % of the cells displayed red fluorescence at excitation/emission wavelengths of 587/610 nm. qRT-PCR assays were used to detect the expression of miR-375 in these stable cell lines.

### Cell proliferation assay

Cell proliferation was evaluated using a Cell-Counting Kit 8 (CCK8), as described by the manufacturer (Dojindo Molecular Technologies, Inc., Kumamoto, Japan). Two thousands of DU145 or PC-3 cells per well were cultured in 96-well plates and 10 μL of CCK-8 solution was added to each well at the indicated time points after transfection. Cells were further incubated for 2 h at 37 °C in a 5 % CO_2_ incubator. The absorbance was measured at 450 nm with Multiscan FC Microplate Photometer (Thermo Fisher Scientific, Rochester, NY, USA).

### Flow cytometry

Cell apoptosis was detected using Annexin V-PE Apoptosis Detection Kit (BD Pharmingen, San Jose, CA, USA). Cells in the logarithmic phase of growth were harvested and washed twice in PBS. Based on the manufacturer’s instruction, 1 × 10^6^ cells were washed twice in PBS before re-suspension in 1X Binding Buffer. 5 μl of PE Annexin V and 5 μl 7-AAD were added and stained on ice for 30 min, followed by adding 400 μl of 1X Binding Buffer to each tube. Stained cells were measured by flow cytometry (FACSCalibur, BD Bioscience, Heidelberg, Germany) using Cell Quest Pro software (BD Bioscience). The data were analyzed using FlowJo9.1 software.

### Western blot

Cells were lysed in RIPA buffer (1 % NP-40, 0.5 % sodium deoxycholate, 0.1 % SDS in PBS). Complete protease inhibitor cocktail (Roche, Indianapolis, IN, USA) was added to lysis buffer before use. Protein concentration was determined by Bio-Rad DC protein assay (Bio-Rad, Hercules, CA, USA). 20ug of total protein from cell lysate was subjected to SDS-PAGE and transferred to nitrocellulose membrane. The membrane was blocked in 5 % non-fat milk in PBS overnight and incubated with primary antibody. After washing for 30 min, secondary goat anti-mouse IgG (Vector Co., Burlingame, CA, USA) was applied to nitrocellulose membrane in TBS-Tween for 1 h. After washing, the proteins of interest were detected using Chemiluminescent HRP Antibody Detection Kit (Denville Scientific, South Plainfield, NJ, USA). Anti-SEC23A polyclonal antibody was purchased from Sigma-Aldrich (St. Louis, MO, USA). Anti-β-tubulin and anti-YAP antibodies were purchased from Cell signaling technology (Danvers, MA, USA). The protein signals were captured using an electrochemiluminescent system (PerkinElmer Life Science, Boston, MA, USA).

### Animal experiments

Female athymic nude mice at eight weeks of age were purchased from the Jackson Laboratory (Bar Harbor, ME). Animals were housed with wood chip bedding in environmentally controlled, clean-air room with a 12-h light–dark cycle and a relative humidity of 50 %. Drinking water and diet were supplied ad libitum. The study was approved by the Institutional Animal Care and Use Committee at the Medical College of Wisconsin.

Mice were inoculated subcutaneously (s.c.) at two sites per mouse with PC-3 cells stably transfected with lentivirus for either miR-375 or empty vector (*n* = 16). Xenografts were inoculated with 2.5 × 10^6^ cells suspended in 200 μL PBS (phosphate-buffered saline) with 50 % Matrigel (BD Biosciences). When tumors reached an average volume of 198 mm^3^, mice were randomly assigned into vehicle control group (0.5 % DMSO in PBS) or 10 mg/kg.bw docetaxel (Cayman Chemicals, Ann Arbor, MI). Mice were administered intraperitoneally (i.p.) once per week for 4 weeks. Mice were weighed and tumor volumes were measured twice a week with digital caliper (volume = π/6*length*width^2^). Experiments were stopped 7 days after final injections. At the end point, mice were euthanized by CO_2_ asphyxiation and tumor tissues were fixed in zinc formalin solution overnight and stored in 70 % ethanol for histopathology evaluation or flash frozen in liquid nitrogen for RNA and protein evaluation.

### Histology and Immunohistochemistry

Mouse xenografts were Zinc formalin fixed for 24–72 h before paraffin processing on a Sakura VIP tissue processor. After paraffin embedding samples, 2 slides were sectioned at 4um with 100um levels separating sections for each xenograft and dried overnight at 45 °C. All slides stained for Cleaved Caspase-3 (Biocare, CP-229) were deparaffinized and subjected to antigen retrieval with citrate buffer pH6 and EDTA pH9 respectively for a total of 40 min. After peroxidase and protein blocking, antibody was incubated at room temperature for 90 min. The standard labelled streptavidin (HRP)-biotin detection technique was used for visualization using 3,3′-diaminobenzidine to detect the perioxidase. Positively stained cells were counted in at least five fields from each area with 400× magnification. The results were expressed as the average of positive cells per five 400× magnifications (approximately corresponding to 0.035 mm2). Differences of means per area between groups were determined by ANOVA using SAS. Differences were considered significant when *P* ˂ 0.05.

### TCGA data analysis in PC patients

TCGA dataset was used to obtain both miRNA and mRNA expressions, when available, from PC and normal tissue samples. All miRNA/mRNA expression data were downloaded from TCGA data matrix (http://tcga- data.nci.nih.gov/tcga/tcgaDownload.jsp). This dataset included 495 PC tumor tissues and 54 partially matched normal tissues. To prevent duplicates, when there was more than one dataset for a particular sample, average values were used. The expression values were pre-processed and normalized according to ‘level 3’ specifications of TCGA (for details, see http://cancergenome.nih.gov/).

### miRNA target prediction and statistical analysis

The miRWalk database was used to identify potential targets of miRNA-375. 3′-UTRs with a seed match of at least 7 bases and a *p*-value <0.05 were searched using three database algorithms: TargetScan, PITA and DIANA-mT. The predicted miR-375 targets are listed in Additional file 1: Table S1. Statistical data were presented as the mean ± standard deviation (SD). Student’s *t*-test was performed for comparisons using Excel. A value of *p* < 0.05 was considered statistically significant.

## Results

### miR-375 is upregulated in tumor tissues and is induced by docetaxel in PC cell lines

**Fig. 1:**
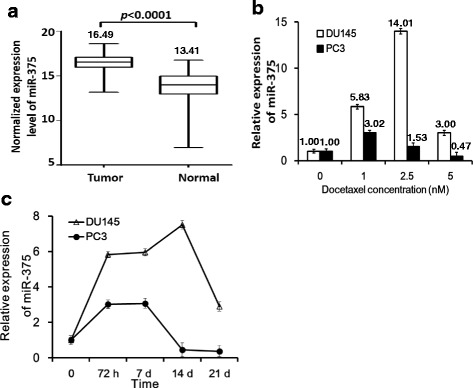
miR-375 expression in PC tissues and its response to docetaxel PC cell lines. **a** Overexpression of miR-375 in PC tissues. Normalized miR-375 expression levels were downloaded from TCGA. Compared to normal prostate tissues, expression levels of miR-375 in PC tissues are significantly higher. Number on the top of each box plot is mean value of miR-375 expression. **b** Response of miR-375 to docetaxel in PC cells. miR-375 expression levels were measured by qRT-PCR under different concentrations of docetaxel. The result shows dose-dependent upregulation of miR-375. Number on the top of each bar plot is mean fold change of miR-375 expression when compared to controls. **c** Dynamic expression changes of miR-375 during and after docetaxel treatment. 1 nM docetaxel was used until 72 h. Expression level was measured at days 0, 3, 7, 14, and 21, respectively. miR-375 expression was upregulated during treatment and gradually reduced after removal of docetaxel. MicroRNA expression values were rescaled relative to the blank control

We previously reported a significant association of elevated plasma miR-375 expression with poor overall survival in mCRPC stage. Since some of these patients in this cohort had also received docetaxel chemotherapy [13] we tested if miR-375 is involved in the development of docetaxel resistance and may have impacted the overall survival. We first compared miR-375 expression levels between PC tissues and normal prostate tissues in TCGA dataset. We observed 8.45-fold increase of miR-375 expression in PC tissues than in normal tissues (Fig. 1a). To further study the impact of docetaxel chemotherapy on the expression of miR-375 in PC cells, we then treated DU145 and PC-3 cells with docetaxel in different concentrations (1 nM, 2.5 nM, and 5 nM) for 72 h. qRT-PCR analysis showed that miR-375 expression was significantly upregulated after docetaxel treatments (Fig. 1b). Treatment of the cells with docetaxel at concentrations of 1 and 2.5 nM, induced 5.83- and 14.01-fold increase in miR-375 expression in DU145 cells, respectively, and 3.02- and 2.53- fold increase in PC-3 cells, respectively.

To evaluate the long term effects of docetaxel on miR-375 expression, DU145 and PC-3 cells were cultured with 1 nM docetaxel. After 72 h of treatment, we removed docetaxel from the culture media and continued the cell culture until day 21. We performed qRT-PCR and measured expression levels of miR-375 at days 0, 3, 7, 14, and 21, respectively. Our data showed docetaxel-associated progressive upregulation of miR-375 expression in both DU145 and PC-3 cells. The miR-375 expression level reached a peak at day 7 in PC-3 cells, or day 14 in DU145 cells, and gradually declined until day 21 (Fig. 1c). These findings suggest that the expression level of miR-375 in PC is docetaxel-dependent. Because miR-375 level keeps increase (in PC-3 cells) or remain unchanged (in DU145) for several days after docetaxel removal, this docetaxel-induced miR-375 upregulation was more likely to be an indirect effect.

### miR-375 contributes to docetaxel resistance in PC cell lines

**Fig. 2:**
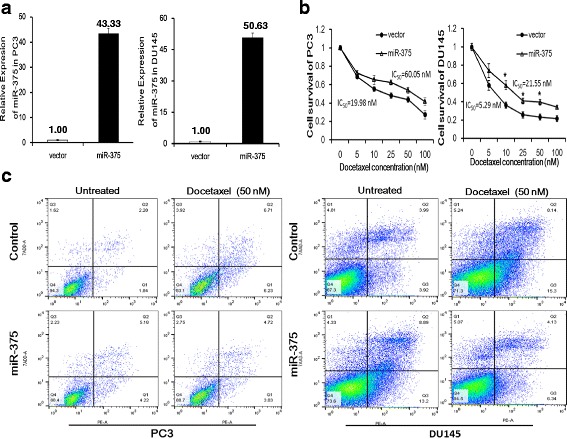
miR-375 induced docetaxel resistance in PC cells. **a** After miR-375 lentivirus transfection, both DU-145 and PC-3 cell lines show stably high level expression. **b** PC cells were treated with different concentrations of docetaxel for 48 h. Cell proliferation assay shows that cells with miR-375 transfection are more tolerant to docetaxel toxic effect. **c** 24 h after miR-375 transfection, cells were treated with docetaxel (50 nM) for another 24 h. Flow cytometry analyses show that high levels of miR-375 protect cells from apoptosis in both PC-3 and DU145 cell lines. Fraction of apoptotic cells are shown in lower right corners

To determine the effect of miR-375 on proliferation of PC cell lines, PC-3 and DU145 cells were transiently transfected with 200 nM miR-375 mimic. Cell proliferation was assayed by the CCK-8 method. The proliferation rate of PC-3 and DU145 cells at 72 h after transfection was significantly reduced (*P* = 0.003 and *P* = 0.001, respectively) when compared to that of miRNA negative control (see Additional file 2: Figure S1). To evaluate the involvement of miR-375 in regulation of docetaxel sensitivity, we stably transfected a miR-375 expression lentivirus and control vector into PC cell lines. As shown in Fig. 2a, qRT-PCR analysis confirmed the high level expression of miR-375 after transfection. To determine the sensitivity of PC cells to docetaxel, we performed a cell proliferation and cytotoxicity assay (CCK-8) at 48 h using different docetaxel doses (0, 5, 10, 25, 50 and 100 nM). As shown in Fig. 2b, miR-375 transfected cells consistently showed higher survival rate (therefore, docetaxel resistance) in both PC-3 and DU145 cell lines. The IC_50_ values were increased in PC-3 and DU145 with miR-375 overexpression (60.05 ± 1.63 and 21.55 ± 0.96 nM, respectively) when compared to vector controls (19.98 ± 1.54 and 5.29 ± 0.57 nM, respectively) (PC-3, *P* = 0.021; DU145, *P* = 0.033). To determine whether the effects of miR-375 on cell proliferation were related to apoptosis, we conducted an apoptosis assay by flow cytometry. We first transfected PC cells with 200 nM miR-375 mimic for 24 h and then treated the cells with docetaxel (50 nM) for another 24 h. Our results showed that ectopic miR-375 expression decreased docetaxel-induced cellular apoptosis. For example, when comparing post- to pre-docetaxel treatment, PE-labeled Annexin V positive cell fraction increased from 3.92 to 15.3 % in control DU145 cells, while decreased from 13.2 to 6.34 % in miR-375 transfected DU145 cells. Similar data were also observed in PC-3 cell line (Fig. 2c). Cells with miR-375 overexpression is clearly resistant to docetaxel treatment in PC cells.

### miR-375 is involved in the development of docetaxel resistance in PC cells grown as xenograft tumors in nude mice

**Fig. 3:**
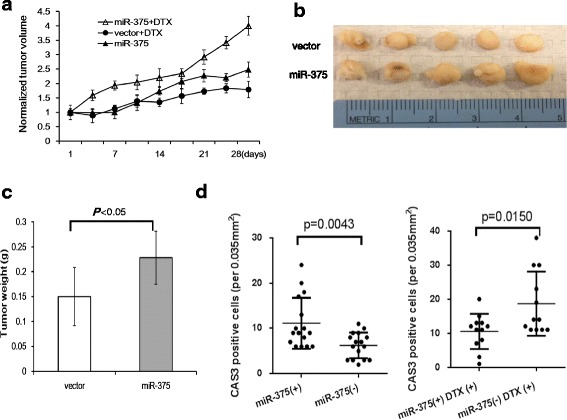
Effect of miR-375 on docetaxel resistance in xenograft mice. Nude mice were inoculated with PC-3 cells transfected miR-375 or control vector to allow tumor development. Mice were intraperitoneally injected with 10 mg/kg docetaxel once a week. **a** Tumor volumes in the docetaxel-treated mice are increased with time, in particular the xenograft tumors overexpressing miR-375. **b** Xenografts with empty control vector were significantly smaller than the miR-375 overexpression group. Representative tumors from each group are shown. **c** Tumor weights at the end of the treatment period (28 days after first docetaxel injection) were significantly higher in miR-375 group. Data are represented as the mean ± SD. (*n* = 6, *P* <0.05). **d** Caspase-3 immunohistochemistry analyses of xenograft tumors with empty control vector or miR-375 lentivirus vector after docetaxel treatment. Under docetaxel (DTX) treatment, less apoptotic cells were observed in miR-375 overexpressed xenograft tumors than miR-375 vector controls

To further test for the involvement of miR-375 in docetaxel resistance, we injected the stably transfected cell lines grown as xenograft tumors in athymic nude mice. We observed consistently higher tumor volume in tumors with miR-375 overexpression than tumors with empty vector under docetaxel administration, in particular, at day 28 after initiation of docetaxel treatment (Fig. 3a). At the end of experiment period, the mean wet weight of tumors was significantly higher in miR-375 transfected group than in empty vector control group (*p* < 0.05) (Fig. 3b and c). Clearly, the higher expression of miR-375 is also associated with docetaxel resistance in vivo.

To further evaluate the potential effect of miR-375 expression on death in vivo, we tested the presence of active caspase-3 by immunohistochemistry in xenograft tumors. Caspase-3 is a cytosolic enzyme that is activated only in cells committed to undergo apoptosis. The activation of caspase-3 precedes the development of the classical morphological features of apoptosis. The immunostaining analysis showed that miR-375 overexpression tumors had significantly higher levels of activated caspase-3 positive cells than empty vector control tumors (11.13 ± 5.64 vs. 6.25 ± 2.84, *P* = 0.0043). However, miR-375 overexpression tumors treated with docetaxel showed lower levels of caspase-3 positive cells than empty vector tumors treated with docetaxel (10.5 ± 5.16 vs. 18.67 ± 9.40, *P* = 0.0150) (Fig. 3d). This conflicting result demonstrates that under the condition of docetaxel treatment the anti-apoptotic effect of miR-375 overexpression overshadows its growth inhibition. This was in line with the in vitro experiments. Representative immunostainings are shown in Additional file 3: Figure S2.

### miR-375 regulates *SEC23A* and *YAP1* expression

**Fig. 4:**
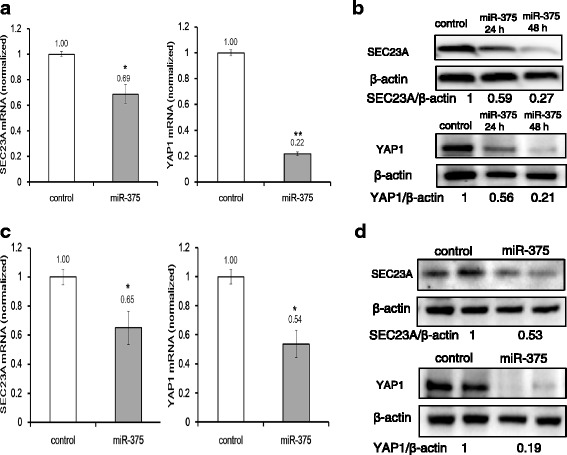
Expression of miR-375 and its target genes (*YAP1* and *SEC23A*) in PC cells or xenograft tumors. **a** qRT-PCR analysis showed that *YAP1* and *SEC23A* expressions were downregulated after miR-375 transfection in PC-3 cells. * indicates statistically significant reduction. **b** Western blots showed suppression of YAP1 and SEC23A proteins in the miR-375-transfected PC-3 cells. **c** qRT-PCR analysis showed that *YAP1* and *SEC23A* expression levels were lower in xenografts overexpressing miR-375. **d** Western blots showed suppression of YAP1 and SEC23A proteins in xenograft tumors overexpressing miR-375. β-actin served as a loading control to normalize protein signal intensity

miR-375 has been shown to target *YAP1* in lung cancer cells [18] and also *SEC23A* at its 3′-UTR in PC cell lines to regulate cell growth [16]. To investigate whether miR-375 directly regulates *YAP1* or *SEC23A* expression, we transfected PC cell lines with a miR-375 mimic for 48 h, and evaluated the *SEC23A* and *YAP1* mRNA expression by qRT-PCR. Transfection with miR-375 in PC-3 cells resulted in significant reduction of *SEC23A* and *YAP1* mRNA expression with 31 and 78 % decreases, respectively (Fig. 4a). Furthermore, Western blotting of PC-3 cells expressing miR-375 showed a significant reduction in *SEC23A* and *YAP1* protein levels (Fig. 4b). We also evaluated if elevated miR-375 impacts the transcription levels of its target genes in vivo in PC xenograft tumors. Both qRT-PCR and Western blot analysis showed that miR-375 transfection caused significant reduction of *SEC23A* and *YAP1* mRNA expression (Fig. 4c), and protein (Fig. 4d) in xenograft tumors grown in mice.

### miR-375 expression is inversely correlated with *SEC23A* and *YAP1* in PC tissues

**Fig. 5:**
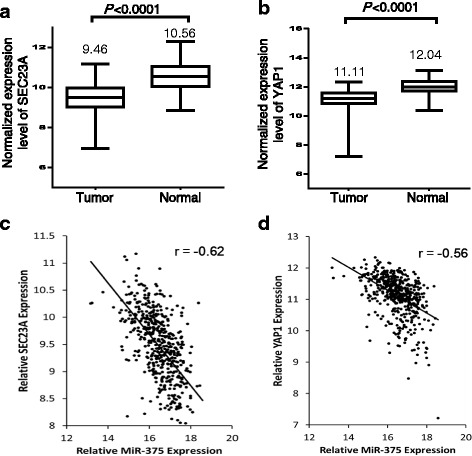
Inverse relationship of expression levels between miR-375 and its target genes (*YAP1* and *SEC23A*) in TCGA dataset. **a** Compared to normal prostate tissues, expression levels of *SEC23A* were significantly lower in PC tissues. **b** Compared to normal prostate tissues, expression levels of *YAP1* were significantly lower in PC tissues. **c** Negative correlation of miR-375 with *SEC23A* expression (Pearson’s correlation r = −0.62, *P* < 0.0001). **d** Negative correlation of miR-375 with *YAP1* expression (Pearson’s correlation r = −0.56 *P* < 0.0001)

To evaluate if miR-375 may target *SEC23A* and *YAP1* in patient-derived PC tissues, we first compared *SEC23A* and *YAP1* expression levels between PC tissues and normal prostate tissues in TCGA data. The results showed that *SEC23A* and *YAP1* levels were significantly decreased in PC tissues when compared to normal tissues (*p* < 0.0001) (Fig. 5a and b). We then performed a correlation analysis between the expression levels of miR-375 and its two target genes. This analysis demonstrated that the levels of *SEC23A* and *YAP1* were inversely correlated with miR-375 expression level (*r* = −0.62 for *SEC23A* and −0.56 for *YAP1*, *p* < 0.05) (Fig. 5c and d). These data indicate that the inhibitory effects of miR-375 on *SEC23A* and *YAP1* are also relevant in clinical samples in PC patients.

## Discussion

Docetaxel is a common chemotherapeutic agent used to treat multiple malignancies including mCRPC stage. However, there are no markers of response/resistance known for docetaxel. Molecular profiling of miRNAs may help in developing companion diagnostics in the clinic to identify subgroups of patients destined to respond to this commonly used chemotherapy and also to understand the molecular mechanisms of chemo-resistance [9, 10]. miRNA regulation of gene expression has been shown to play an important role in the development of cancer drug resistance. By directly targeting protein-coding genes, miRNAs are able to inhibit genes that are necessary for signaling pathways or drug-induced apoptosis that render cells resistance^20^. Multiple miRNAs have been found to be critical in the control of cancer drug resistance in PC [2, 8]. In this study, we examined the influence of docetaxel on miR-375 expression and its target genes using in vitro and in vivo assays. Our results suggest that miR-375 contributes to the development of chemo-resistance in PC.

Previous studies showed that miR-375 is down-regulated in many types of cancer [15], and therefore, miR-375 is believed to act as a critical tumor suppressor by targeting important oncogenes, and modulating cancer-related processes such as cell proliferation, apoptosis, invasion and migration, metastasis and autophagy [15, 19–22]. However, miR-375 is also reported to be upregulated in some cancer types including PC [15] and higher expression of miR-375 is associated with PC stage and lymph node metastasis [23]. We have previously shown that elevated miR-375 is significantly associated with poor overall survival in mCRPC patients [13]. Surprisingly, the current study demonstrated consistent growth inhibition of elevated miR-375 in PC cell lines. This unexpected growth inhibition seems contradictory to poor overall survival in patients with higher level of miR-375. However, further in vitro and in vivo study showed that higher miR-375 also rendered resistance to docetaxel treatment. This conflicting result suggests that while high miR-375 causes cell growth arrest the treatment with docetaxel may create an environment that favors cell growth, possibly through inhibiting cell apoptosis. The anti-apoptotic effect clearly overweighs the cell growth inhibitory effect. Therefore, high level of miR-375 is expected to promote tumor growth and reduce overall survival during docetaxel treatment.

To further investigate a potential role of miR-375 in docetaxel resistance, we performed qRT-PCR and western blot analysis, and confirmed the association of elevated miR-375 with decreased expression of two target genes, *SEC23A* and *YAP1*. This inverse relationship is also clearly evidenced in PC tissues of TCGA dataset. It has been reported that *YAP* is a transcriptional coactivator and functions as the major effector of the Hippo tumor suppressor pathway, which controls cell growth, tissue homeostasis, and organ size [24]. Inhibition of *YAP1* expression greatly reduces detachment-induced apoptosis [25]. *YAP1* and *AR* co-localize and interact with each other predominantly within cell nuclei by an androgen-dependent mechanism in a hormone naive and an androgen-independent mechanism in mCRPC cells [26]. The role of *YAP1* in PC, however, is not limited in AR sensitive cells. A new study [27] has shown an important role of *YAP1* in other PC cell lines. Compared to low expression in LnCap cells, *YAP1* is highly expressed in more invasive cell lines including PC3 and DU145. Ectopical expression of miR-375 significantly reduced the *YAP1* expression of these cell lines both in mRNA and protein level. This study revealed that *ZEB1-miR-375-YAP1* pathway regulates epithelial plasticity in PC.

A cumulative evidence has shown that acquisition of chemo-resistance in cancer cells is accompanied by epithelial-mesenchymal transition (EMT) phenotype. miR-375 is inversely correlated with EMT signature [27]. Knockdown of *YAP1* phenocopied miR-375 overexpression. Another study shows that miR-375 mediated chemo-resistance in cervical cancer by facilitating EMT [17]. *SEC23A*, a member of the coatomer protein complex II (COPII) machinery, is instrumental in altering the tumor cell secretome in various cancers [28, 29]. The gene was reported to regulate the secretion of metastasis-suppressive proteins [30] and is involved in anti-tumorigenesis. Ectopic expression of *SEC23A* reduces growth properties while inhibition of *SEC23A* enhances the growth properties of PC cell lines [16]. A recent study shows that SEC23A is a novel target of miR-375 and is significantly downregulated in PC cells and tissues [31]. Potentially, overexpression of miR-375 in PC cells directly inhibits production of *SEC23A*, which prevents the secretion of metastasis-suppressive proteins, thus enabling the metastatic spread and colonization of the cancer cells. Therefore, these experimental evidences strongly support that miR-375 induces PC docetaxel resistance by down-regulating *SEC23A* and *YAP1*.

## Conclusion

We performed cell line and animal-based studies to establish a role for miR-375 in docetaxel resistance. The results from this study suggest that docetaxel may further upregulate miR-375 expression during chemotherapy and confer chemo-resistance by inhibiting expression of *SEC23A* and *YAP1.* This mechanistic basis may explain (at least partially) the development of docetaxel resistance. This study further confirms that miR-375 is not only an important candidate biomarker for clinical outcome prediction but also a key target for future therapeutic drug development with or without targeting *SEC23A* and *YAP1* activity. Further understanding biological role of miR-375 in PC therapeutics will facilitate discovery of new treatment approaches to improve drug efficacy in the patients with advanced PC.

## Additional files

Additional file 1: Table S1. miR-375 targeted genes. (XLSX 400 kb)
Additional file 2: Figure S1. Higher miR-375 suppresses cell proliferation in PC cells. Overexpression of miR-375 by transfection with 200 nM miR-375 mimic inhibited cell growth when compared to miRNA negative control in PC-3 and DU145 cells at 72 h after transfection. (*P* < 0.01). (PPTX 82 kb)
Additional file 3: Figure S2. Representative immunohistochemistry analysis of xenograft tumor tissues. Cells with active caspase-3 were shown in brown color. A, B and C are representative images for a lower fraction of apoptotic cells, a higher fraction of apoptotic cells, and negative controls, respectively. (PPTX 1067 kb)

## Abbreviations

CCK8: Cell-Counting Kit 8
DMSO: Dimethyl sulfoxide
EMT: Epithelial–mesenchymal transition
*GADPH*: Glyceraldehyde-3-phosphate dehydrogenase
mCRPC: Metastatic castrate-resistant prostate cancer
PBS: Phosphate-buffered saline
PC: Prostate cancer
qRT-PCR: Quantitative reverse transcription PCR
RIPA: Radioimmunoprecipitation assay
SDS-PAGE: Sodium dodecyl sulfate polyacrylamide gel electrophoresis
SEC23A: Sec23 homolog A
TCGA: The Cancer Genome Atlas
YAP1: Yes associated protein 1
