# Supplementary figures and images for: miR-375 induces docetaxel resistance in prostate cancer by targeting SEC23A and YAP1
Source: Mol Cancer. 2016 Nov 10;15:70. doi: 10.1186/s12943-016-0556-9 (PMC5105253; doi:10.1186/s12943-016-0556-9)

## Slide 1
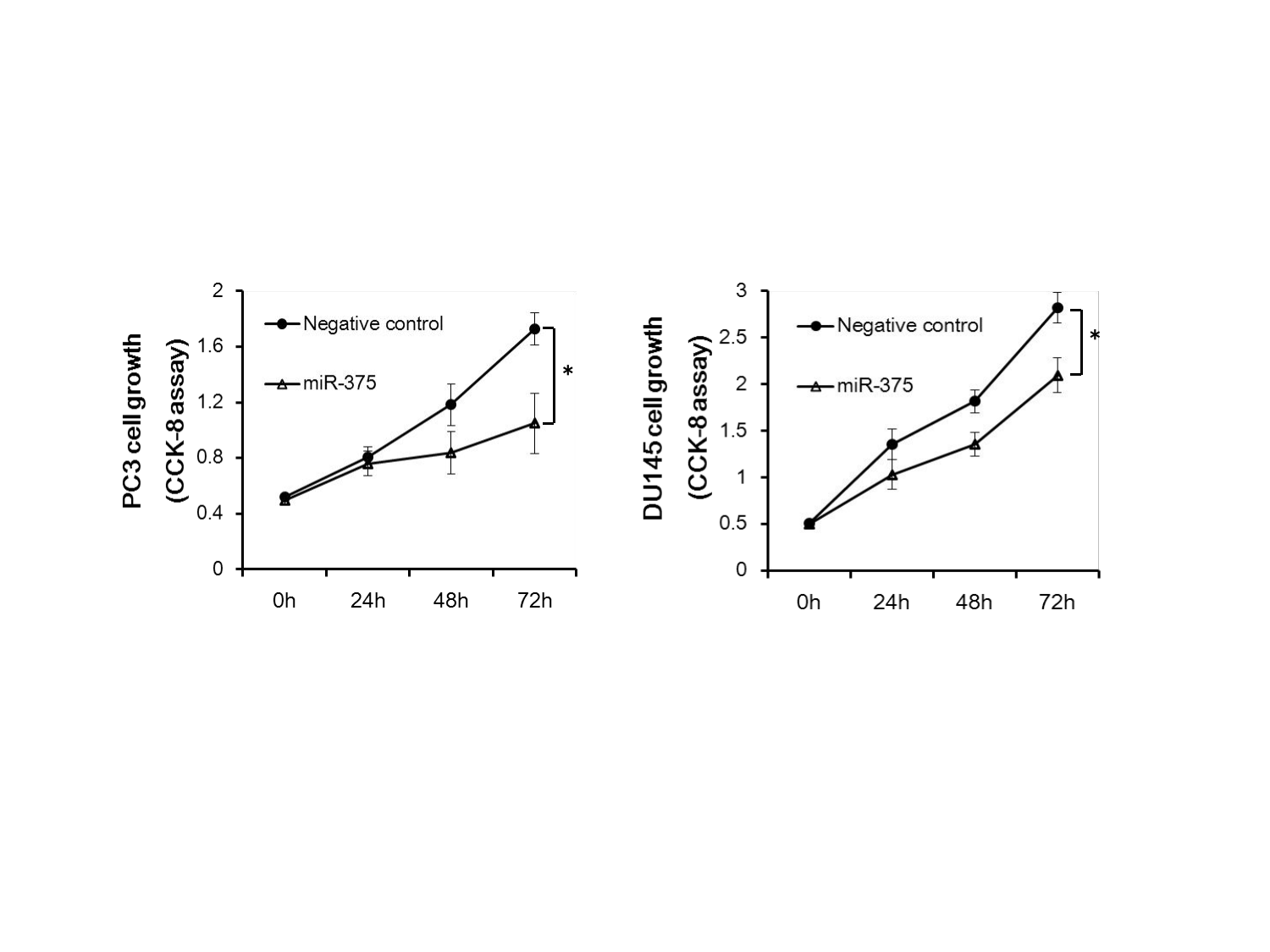

*
*

Supplement: Additional file 2: Figure S1. — Higher miR-375 suppresses cell proliferation in PC cells. Overexpression of miR-375 by transfection with 200 nM miR-375 mimic inhibited cell growth when compared to miRNA negative control in PC-3 and DU145 cells at 72 h after transfection. (P < 0.01). (PPTX 82 kb) [file 12943_2016_556_MOESM2_ESM.pptx]

## Slide 1
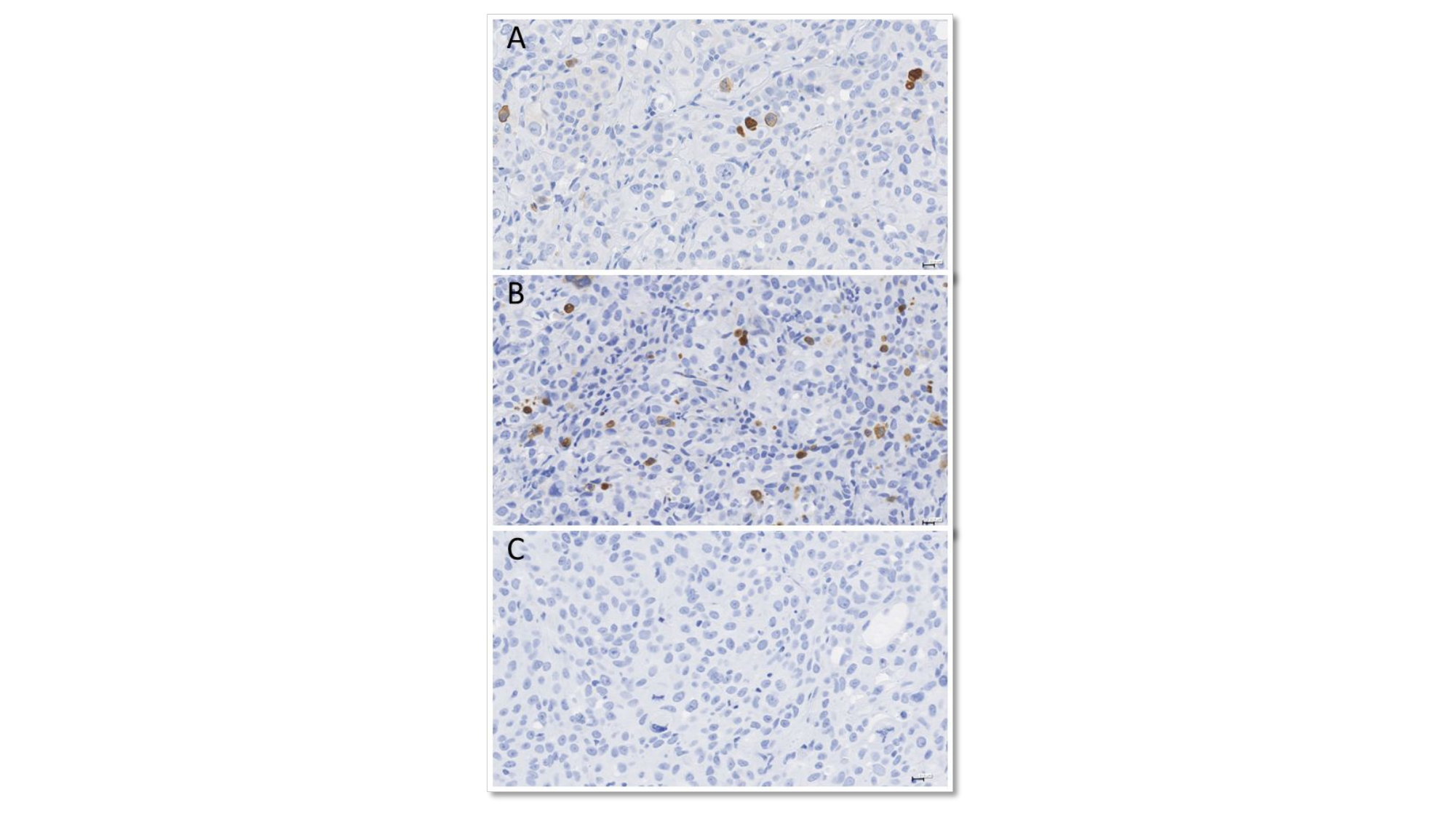

Supplement: Additional file 3: Figure S2. — Representative immunohistochemistry analysis of xenograft tumor tissues. Cells with active caspase-3 were shown in brown color. A, B and C are representative images for a lower fraction of apoptotic cells, a higher fraction of apoptotic cells, and negative controls, respectively. (PPTX 1067 kb) [file 12943_2016_556_MOESM3_ESM.pptx]
